# Oxygen–Glucose Deprivation Promoted Fibroblast Senescence and Collagen Expression via IL11

**DOI:** 10.3390/ijms232012090

**Published:** 2022-10-11

**Authors:** Tongtong Song, Yiwen Gu, Wenting Hui, Xiaoyu Yang, Yanqing Liu, Xia Chen

**Affiliations:** Department of Pharmacology, College of Basic Medical Sciences, Jilin University, Changchun 130012, China

**Keywords:** fibroblasts, senescence, collagen, IL11, IDO1

## Abstract

Cell senescence is one of the most important forms of injury induced by cardiovascular and other ischemic diseases. Fibroblasts are important participants in tissue repair after ischemic injury and the main source of IL11 secretion. However, the roles of oxygen–glucose deprivation (OGD) and IL11 in promoting fibroblast senescence and their regulatory mechanisms remain unclear. This study selected the NIH3T3 and L929 fibroblast cell lines as research objects. We found that OGD could induce the expression of p53, P16, p21, and collagen in fibroblasts. In the condition of OGD, when IL11 intervened, fibroblasts’ senescence and collagen expression were changed. Some studies have found that changes in kynurenine (KYN) metabolism are related to aging diseases, and indoleamine 2,3-dioxygenase 1 (IDO1) is a key rate-limiting enzyme in the KYN metabolic pathway. We found that KYN secretion decreased after OGD increased fibroblast senescence, and inhibition of IL11 promoted IDO1 and increased KYN secretion. These results suggest that OGD may promote fibroblast senescence and collagen expression via IL11 inhibition of the IDO1/KYN metabolic pathway. Therefore, the revealed mechanism of OGD-promoted fibroblast senescence could provide an effective theoretical basis for the clinical treatment of aging-related ischemic diseases.

## 1. Introduction

Cell senescence is an irreversible state of cell cycle arrest. A variety of cellular stress states, such as ischemia and DNA damage, can induce cell senescence [1]. Senescent cells are mainly characterized by an increased volume, increased protein expression of senescence markers p53, p21, and p16, and senescence-related secretory phenotypes, which affect the pathophysiological processes of the heart, liver, and other tissues [2,3]. Fibroblasts are an important participant in tissue repair after ischemic injury. It has been found that ischemia can induce fibroblast senescence, and fibroblasts are also a common cell type for studying cell senescence mechanisms in vitro [4]. After tissue ischemia, under the action of cytokines, fibroblasts can be activated into myofibroblasts. Collagen secreted by fibroblasts and myofibroblasts promotes scar formation in the injured area [5], which helps to maintain the integrity of the tissue structure. However, studies have found that fibroblasts continue to activate after stress-induced senescence, leading to the deposition of the extracellular matrix and damaging tissue function [6]. In addition, the expression of p53 and p21 in senescent fibroblasts is up-regulated, which promotes the release of pro-inflammatory factors, intensifies the inflammatory response, and finally aggravates ischemic injury. Therefore, clarifying the mechanism of ischemia-induced fibroblast senescence can provide an effective theoretical basis for the clinical treatment of senescence-related ischemic diseases, which has important clinical significance. NIH3T3 cells and L929 cells are fibroblast cell lines derived from embryonic connective tissue and dermal connective tissue, respectively. They are common objects to study the mechanism of cell senescence, and also common cell lines to study ischemia-related diseases such as myocardial infarction, liver ischemia injury, and chronic injury area healing. It has been reported that hypoxia activates NIH3T3 and L929 cells and increases the expression of collagen. Studies have also shown that H_2_O_2_, serum starvation, and other conditions can induce stress-induced senescence of NIH3T3 and L929 cells and up-regulate p53, p21, p16, and other senescence markers. H_2_O_2_, DOX, X-ray, D-galactose, RAS, serum starvation, and sodium butyrate can induce stress-induced senescence of NIH3T3 and L929 cells and up-regulate senescence markers such as p53, p21, and p16 [7]. However, the effect of oxygen-glucose deprivation (OGD) on fibroblast cell line senescence and its mechanism have not been clarified.

IL11 is a member of the IL6 cytokine family, mainly derived from stromal cells such as fibroblasts [8]. The expression of IL11 promotes tissue fibrosis [9,10]. Recent studies have found that IL11 is closely related to cell senescence. IL11 is a pro-inflammatory and pro-fibrosis factor. Studies have found that it promotes the aging of lung fibroblasts and induces the secretion and deposition of collagen. This process may be mediated by TGFβ/IL11/MEK/ERK signal pathways and is an effective target for preventing aging-related pulmonary fibrosis [11]. IL11 and HO1 increased in the premature senescence of WI-38 human diploid fetal lung fibroblasts [12]. In the senescence prostate model, it is found that the expression of IL11 is increased, and its level can significantly promote the low-level proliferation response of epithelial and stromal fibroblast types [13]. IL11 may be involved in fibroblast senescence and collagen expression, but its mechanism in OGD-promoted fibroblast senescence remains unclear.

Metabolic abnormality is an important feature of cell senescence. According to the analysis of the cell senescence metabolome, Trp metabolism is involved in the process of cell senescence induction [14]. Mitochondrial dysfunction, metabolic imbalance, and increased reactive oxygen species are important causes and manifestations of cell senescence. Kynurenine (KYN) is a tryptophan metabolite that affects mitochondrial function through the synthesis of NAD^+^ and NADP and plays an antioxidant role in the process of cell senescence [15]. The KYN pathway is the most important in tryptophan metabolism, which is mediated by the key rate-limiting enzyme indoleamine 2,3-dioxygenase 1 (IDO1). It has been found that IDO1 is expressed in cardiomyocytes, fibroblasts, and other cells, but the role of IDO1/KYN in OGD-promoted fibroblast senescence remains unclear.

Therefore, our study hypothesized that OGD promotes IL11 expression in fibroblasts, and IL11 promotes fibroblast senescence and collagen secretion by affecting tryptophan metabolism. In this study, the NIH3T3 and L929 fibroblast cell lines from two different connective tissue sources were used to investigate the role and mechanism of OGD in promoting fibroblast senescence. We used IL11 siRNA or an IL11 supplement to investigate the regulatory mechanism of OGD in promoting fibroblast senescence through IL11, which will provide a theoretical basis for the intervention of ischemia-related aging diseases.

## 2. Results

### 2.1. OGD-Promoted Fibroblast Senescence and Collagen-Related Protein Expression

**Figure 1:**
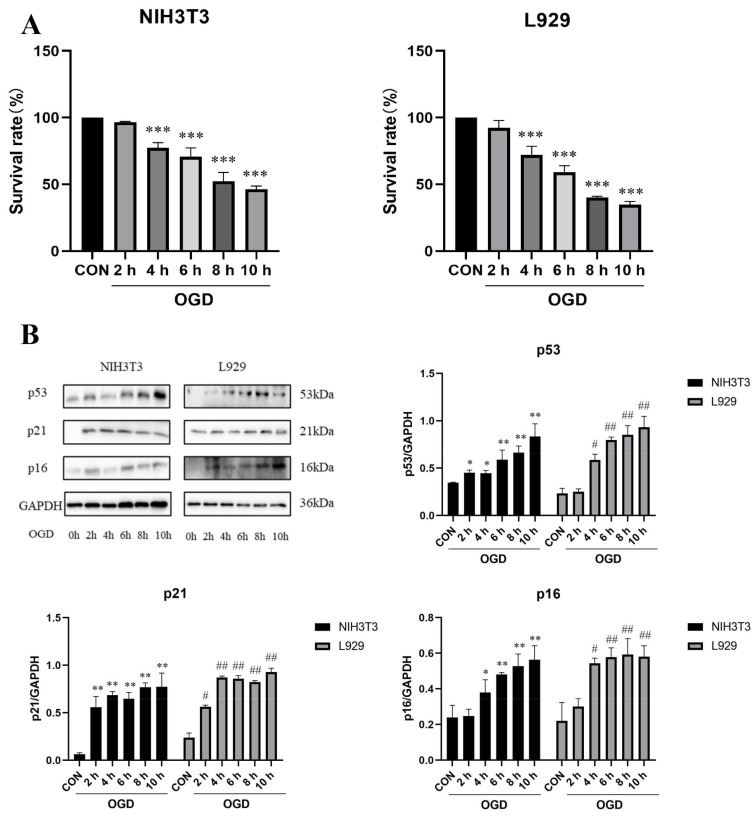
**OGD promoted fibroblast senescence.** (**A**) Survival rates of NIH3T3 and L929 cells under oxygen–glucose deprivation (OGD) conditions for 0 h, 2 h, 4 h, 6 h, 8 h, and 10 h detected by MTT assay. OGD—oxygen–glucose deprivation; CON—control. The results are presented as the means ± SDs, and Student’s t-test was used to determine the *p*-value. * *p* < 0.05, ** *p* < 0.01, and *** *p* < 0.001, compared with CON group. (**B**) Western blot analysis and densitometric analysis of p53, p21, and p16 protein levels in NIH3T3 and L929 cells under OGD conditions for 0 h, 2 h, 4 h, 6 h, 8 h, and 10 h. GAPDH was used as the loading control. OGD—oxygen–glucose deprivation; CON—control. The results are presented as the means ± SDs, and Student’s *t*-test was used to determine the *p*-value. * *p* < 0.05, ** *p* < 0.01, and *** *p* < 0.001, compared with CON group in NIH3T3 cells; ^#^ *p* < 0.05 and ^##^ *p* < 0.01, compared with CON group in L929 cells.

To investigate the effect of oxygen and glucose deprivation on the senescence and collagen expression of fibroblasts, NIH3T3 and L929 cells, which are commonly used to study the mechanism of senescence and collagen, were selected. We first measured the changes in the cell survival rate after 2 h, 4 h, 6 h, 8 h, and 10 h of OGD. The results show that the survival rate of NIH3T3 and L929 cells decreased with time after 4 h, 6 h, 8 h, and 10 h of OGD treatment (Figure 1A). To explore the effect of different action time courses of OGD on the senescence of NIH3T3 and L929 cells, we treated the cells with OGD for 2 h, 4 h, 6 h, 8 h, and 10 h and detected the expression changes of senescence-related marker proteins p53, p21, and p16. The results show that compared with the control group, the expression of the p53, p21, and p16 proteins in NIH3T3 and L929 cells increased significantly after OGD treatment for 4 h, 6 h, 8 h, and 10 h (Figure 1B).

**Figure 2:**
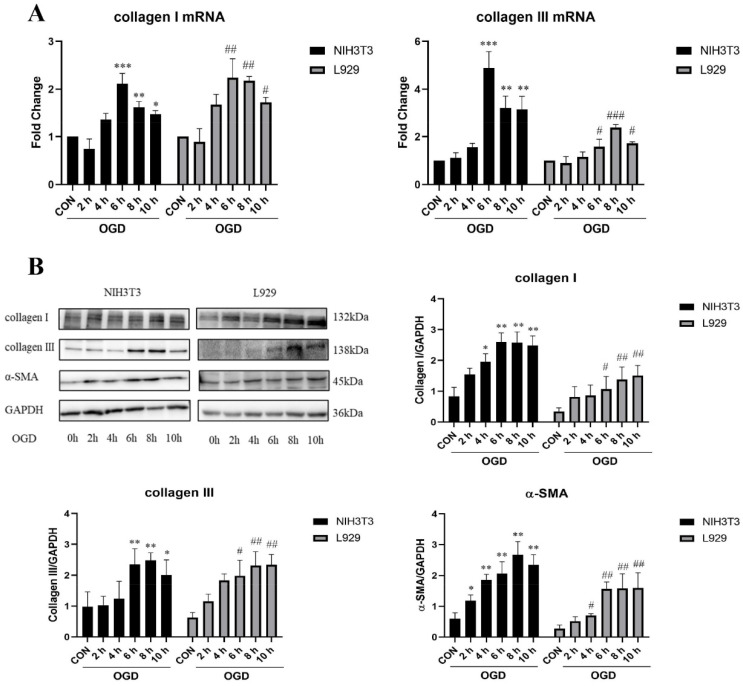
**OGD increased collagen expression.** (**A**) mRNA expression was estimated by RT-PCR for collagen I and collagen III in NIH3T3 and L929 cells under OGD conditions for 0 h, 2 h, 4 h, 6 h, 8 h, and 10 h. (**B**) Western blot analysis and densitometric analysis of collagen I, collagen III, and α-SMA protein levels in NIH3T3 and L929 cells under OGD conditions for 0 h, 2 h, 4 h, 6 h, 8 h, and 10 h. GAPDH was used as the loading control. OGD—oxygen–glucose deprivation; CON—control. The results are presented as the means ± SDs, and Student’s t-test was used to determine the *p*-value. * *p* < 0.05, ** *p* < 0.01, and *** *p* < 0.001, compared with CON group in NIH3T3 cells; ^#^ *p* < 0.05, ^##^ *p* < 0.01, and ^###^ *p* < 0.001, compared with CON group in L929 cells.

To further detect the dynamic changes in collagen expression in NIH3T3 and L929 cells after different treatment periods of OGD, we detected the changes in collagen mRNA and protein levels. The results show that compared with the control group, the mRNA levels of collagen I and collagen III in NIH3T3 and L929 cells increased significantly after OGD treatment for 6, 8, and 10 h (Figure 2A). Collagen I, collagen III, and α-SMA protein expression increased significantly (Figure 2B). In conclusion, OGD can promote senescence and collagen expression in fibroblasts.

### 2.2. OGD Promotes Fibroblast Senescence and Collagen Expression through IL11

**Figure 3:**
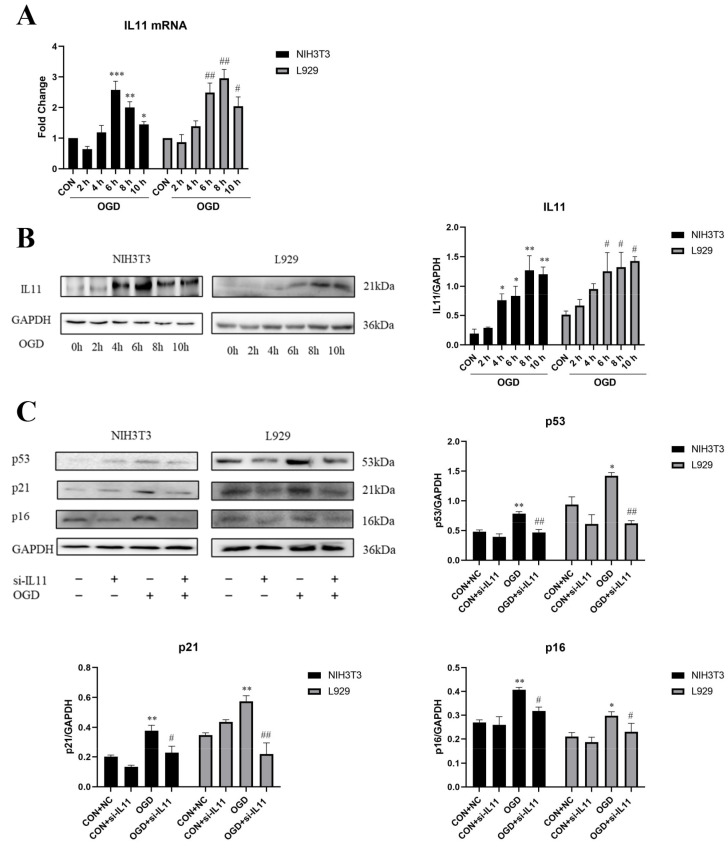
**Interfering with IL11 inhibited fibroblast senescence.** (**A**) mRNA expression estimated by RT-PCR for IL11 in NIH3T3 and L929 cells. (**B**) Western blot analysis and densitometric analysis of IL11 protein levels in NIH3T3 cells and L929 cells; GAPDH was used as the loading control. OGD—oxygen–glucose deprivation; CON—control. The results are presented as the means ± SDs, and Student’s *t*-test was used to determine the *p*-value. * *p* < 0.05, ** *p* < 0.01, and *** *p* < 0.001, compared with CON group in NIH3T3 cells; ^#^ *p* < 0.05 and ^##^ *p* < 0.01, compared with CON group in L929 cells. (**C**) Western blot analysis and densitometric analysis of IL11, p53, p21, and p16 protein levels after inhibiting IL11 with siRNA in NIH3T3 and L929 cells.

**Figure 4:**
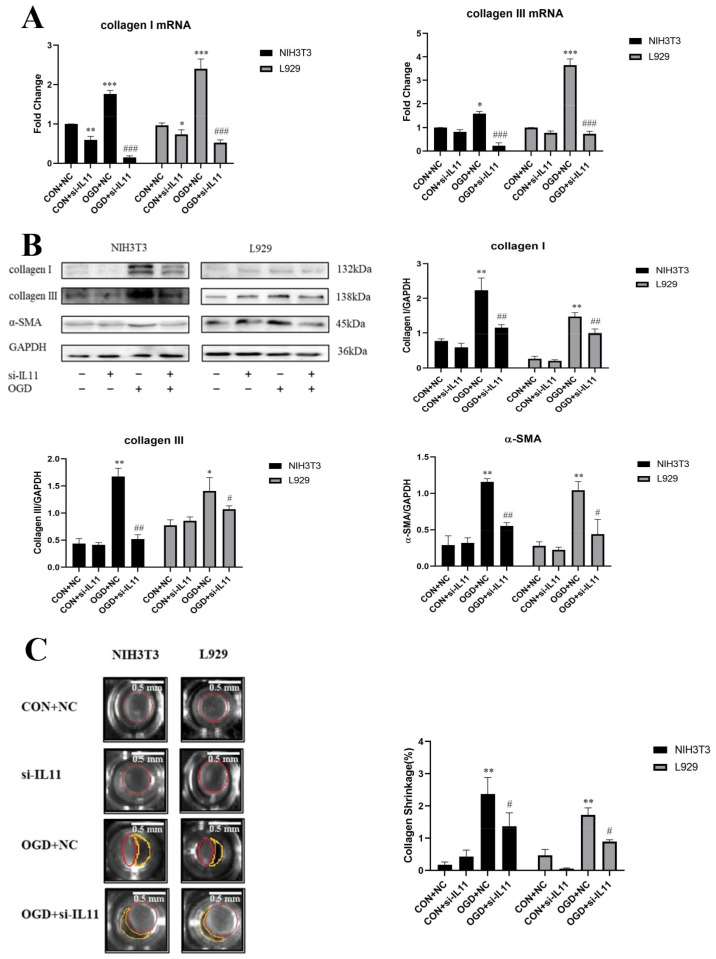
**Interfering with IL11 inhibited fibroblast collagen expression.** (**A**) mRNA expression was estimated by RT-PCR for collagen I and collagen III after inhibiting IL11 with siRNA after OGD-induced NIH3T3 and L929 cell senescence. (**B**) Western blot analysis and densitometric analysis of collagen I, collagen III, and α-SMA protein levels after inhibiting IL11 with siRNA in OGD- induced NIH3T3 and L929 cell senescence; GAPDH was used as the loading control. (**C**) Quantification of gel contraction and representative image of collagen gel containing OGD-induced senescent NIH3T3 and L929 cells with IL11 inhibited. OGD+NC—oxygen–glucose deprivation + negative control; CON+NC—control + negative control. The results are presented as the means ± SDs, and Student’s *t*-test was used to determine the *p*-value. * *p* < 0.05, ** *p* < 0.01, and *** *p* < 0.001, compared with CON+NC group; ^#^ *p* < 0.05, ^##^ *p* < 0.01, and ^###^ *p* < 0.001, compared with OGD+NC group.

To determine the mechanism by which OGD promotes fibroblast senescence and collagen expression, we detected the changes in IL11 expression in NIH3T3 and L929 cells under different OGD treatment periods. The results show that IL11 mRNA and protein expression levels were up-regulated in NIH3T3 and L929 cells after OGD treatment for 6, 8, and 10 h (Figure 3A,B). Based on the results of the cell survival rate, IL11, collagen, and senescence-related protein expression, OGD induction for 6 h was selected as the experimental condition for subsequent verification. To determine whether OGD promotes fibroblast senescence and collagen expression via IL11, we suppressed IL11 expression by transfecting IL11 siRNA. Under the condition of OGD, inhibition of IL11 in NIH3T3 and L929 cells significantly decreased the expression of senescence-related marker proteins p53, p21, and p16 (Figure 3C) and the mRNA and protein expression of collagen I and collagen III (Figure 4A,B) compared with the OGD group. The collagen contraction assay showed that inhibition of IL11 resulted in an OGD-induced reduction in collagen contraction in NIH3T3 and L929 cells (Figure 4C). These results suggest that inhibition of IL11 could inhibit OGD-promoted senescence and collagen expression in fibroblasts.

**Figure 5:**
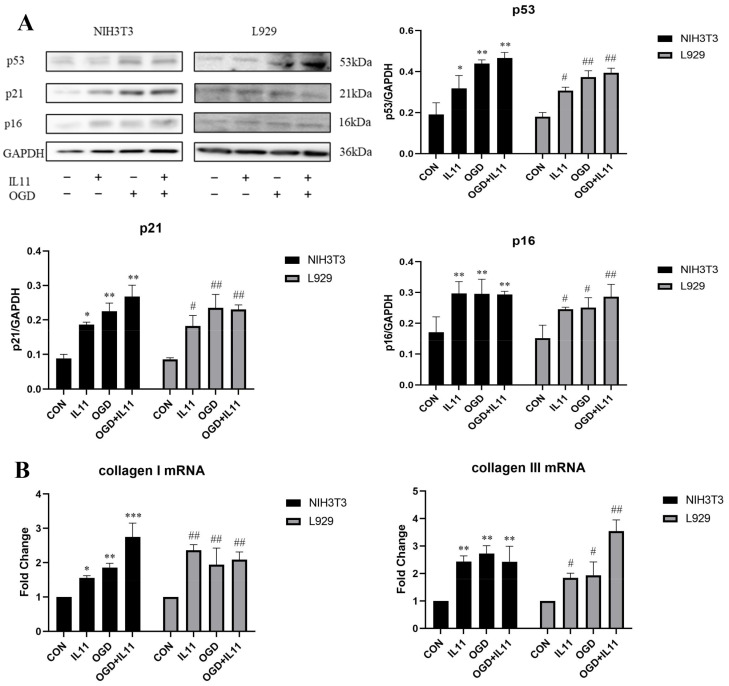
**rmIL11 promoted fibroblast senescence and collagen expression.** (**A**) Western blot analysis and densitometric analysis of p53, p21, and p16 protein levels after supplementing rmIL11 in OGD- induced NIH3T3 and L929 cell senescence. (**B**) mRNA expression estimated by RT-PCR for collagen I and collagen III after supplementing rmIL11 in NIH3T3 cells and L929 cells. OGD—oxygen–glucose deprivation; CON—control. The results are presented as the means ± SDs, and Student’s *t*-test was used to determine the *p*-value. * *p* < 0.05, ** *p* < 0.01, and *** *p* < 0.001, compared with CON group in NIH3T3 cells; ^#^ *p* < 0.05 and ^##^ *p* < 0.01, compared with CON group in L929 cells.

**Figure 6:**
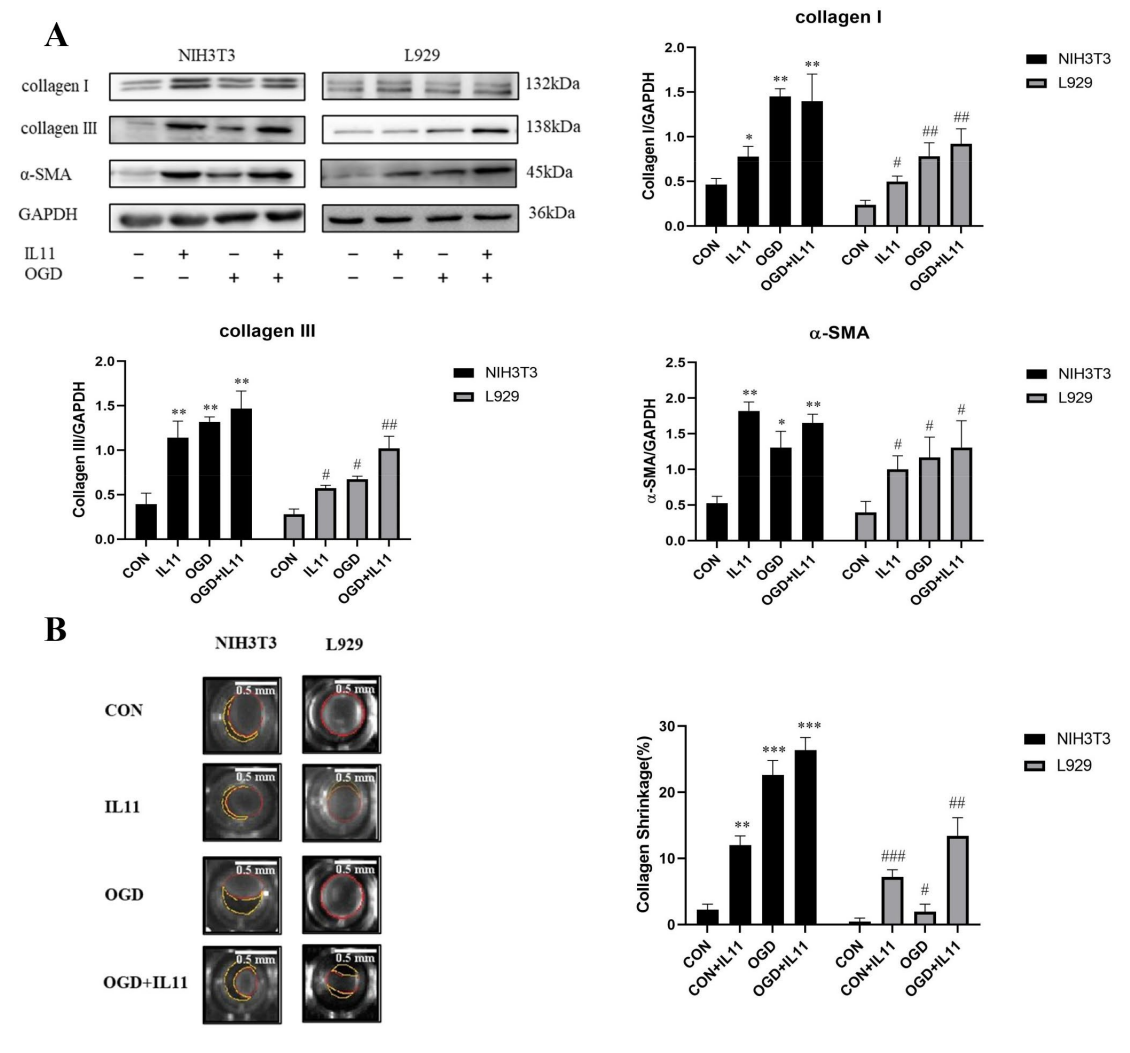
**rmIL11 promoted fibroblast collagen expression.** (**A**) Western blot analysis and densitometric analysis of collagen I, collagen III, and α-SMA protein levels after supplementing rmIL11 in NIH3T3 and L929 cells; GAPDH was used as the loading control. (**B**) Quantification of gel contraction and representative image of collagen gel containing NIH3T3 and L929 cells. OGD—oxygen–glucose deprivation; CON—control. The results are presented as the means ± SDs, and Student’s t-test was used to determine the *p*-value. * *p* < 0.05, ** *p* < 0.01, and *** *p* < 0.001, compared with CON group in NIH3T3 cells; ^#^ *p* < 0.05, ^##^ *p* < 0.01, and ^###^ *p* < 0.001, compared with CON group in L929 cells.

We directly administered recombinant mouse IL11 to investigate the effect of IL11 supplementation on OGD-promoted senescence and collagen expression in NIH3T3 and L929 cells. Western blot analysis showed that IL11 supplementation increased OGD-promoted expression of p53, p21, and p16 proteins in NIH3T3 and L929 cells (Figure 5A). This suggests that IL11 supplementation promotes OGD-promoted senescence of NIH3T3 and L929 cells. Similarly, after IL11 supplementation, the mRNA (Figure 5B) and protein expression levels of collagen I and collagen III increased (Figure 6A), and collagen contraction increased (Figure 6B). Through the above experiments, we proved that IL11 supplementation can promote the senescence and collagen expression of NIH3T3 and L929 cells induced by OGD. In general, it is proved that OGD promoted fibroblast senescence and collagen expression through IL11.

### 2.3. OGD Inhibits Fibroblast IDO1/KYN

**Figure 7:**
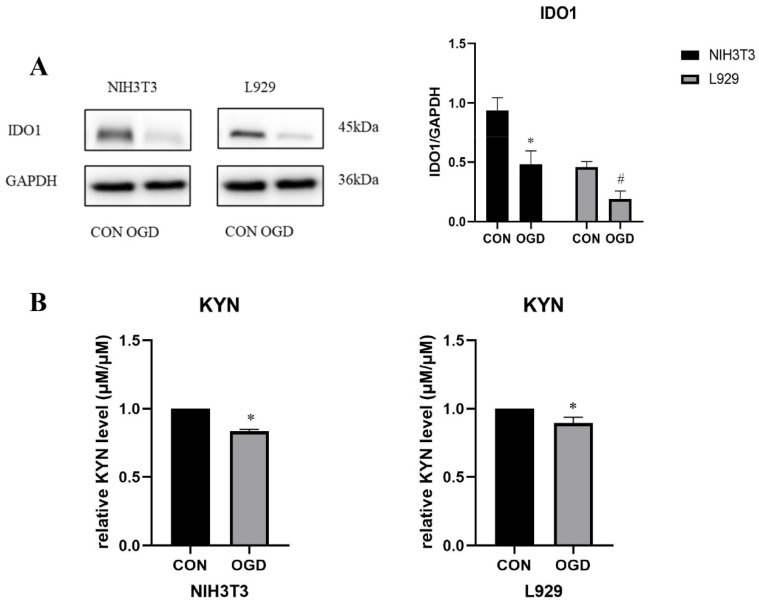
**OGD inhibits fibroblast IDO1/KYN.** (**A**) Western blot analysis and densitometric analysis of indoleamine 2,3-dioxygenase 1 (IDO1) protein levels in NIH3T3 and L929 cells; GAPDH was used as the loading control. The results are presented as the means ± SDs, and Student’s *t*-test was used to determine the *p*-value. * *p* < 0.05, compared with CON group in NIH3T3 cells; ^#^ *p* < 0.05, compared with CON group in L929 cells. (**B**) The relative kynurenine (KYN) level was detected by Ehrlich assay in NIH3T3 and L929 cells. OGD—oxygen–glucose deprivation; CON—control. The results are presented as the means ± SDs, and Student’s t-test was used to determine the *p*-value. * *p* < 0.05, compared with CON group.

To determine the effect of OGD on IDO1/ KYN in fibroblasts, we detected the changes in IDO1/ KYN in NIH3T3 and L929 cells under OGD. The results show that the expression of IDO1 was down-regulated in NIH3T3 and L929 cells under OGD (Figure 7A). The Ehrlich test results show that KYN expression decreased in NIH3T3 and L929 cells under OGD conditions (Figure 7B). The expression of IDO1 and its mediated kynurenine metabolites in NIH3T3 cells changed significantly.

### 2.4. OGD Inhibits the Expression of IDO1/KYN in Fibroblasts via IL11

**Figure 8:**
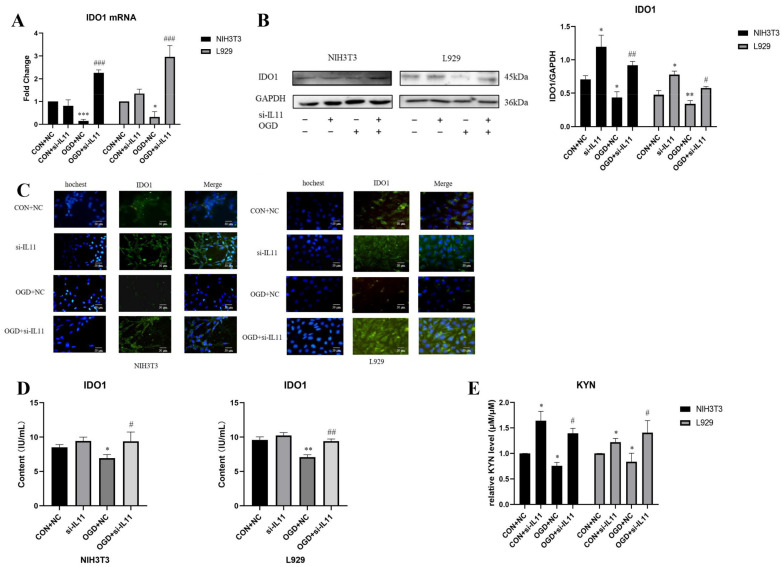
**Interfering with IL11 promoted the expression of IDO1.** (**A**) mRNA expression was estimated by RT-PCR for IDO1 after inhibiting IL11 in NIH3T3 and L929 cells. (**B**) Western blot analysis and densitometric analysis of IDO1 protein levels after inhibiting IL11 in NIH3T3 and L929 cells; GAPDH was used as the loading control. (**C**) Representative immunocytochemistry staining of IDO1 and cell nuclei (Hoechst) after inhibiting IL11 in NIH3T3 and L929 cells. (**D**) IDO1 content estimated by ELISA from treated cell lysate. (**E**) The relative KYN level was detected by Ehrlich assay after inhibiting IL11 in NIH3T3 and L929 cells. OGD+NC—oxygen–glucose deprivation + negative control; CON+NC—control + negative control. The results are presented as the means ± SDs, and Student’s t-test was used to determine the *p*-value. * *p* < 0.05, ** *p* < 0.01, and *** *p* < 0.001compared with CON+NC group; ^#^ *p* < 0.05, ^##^ *p* < 0.01, and ^###^ *p* < 0.001, compared with OGD+NC group.

To determine the regulatory effect of IL11 on IDO1 and KYN, we inhibited IL11 using siRNA and detected the changes in IDO1 by RT-PCR, Western blot, and immunofluorescence. The results show that the mRNA (Figure 8A) and protein expression levels of IDO1 in NIH3T3 and L929 cells increased under OGD conditions (Figure 8B,C). The ELISA kit test results show that after inhibiting IL11, the content of IDO1 in NIH3T3 and L929 cells increased under OGD conditions (Figure 8D). The Ehrlich test results show that after IL11 inhibition, KYN expression in NIH3T3 and L929 cells increased under OGD conditions (Figure 8E). The expression of IDO1 and its mediated kynurenine metabolites in NIH3T3 cells changed significantly. The above results show that interference with IL11 could partially antagonize the inhibitory effect of OGD on the expression of IDO1/KYN in fibroblasts.

**Figure 9:**
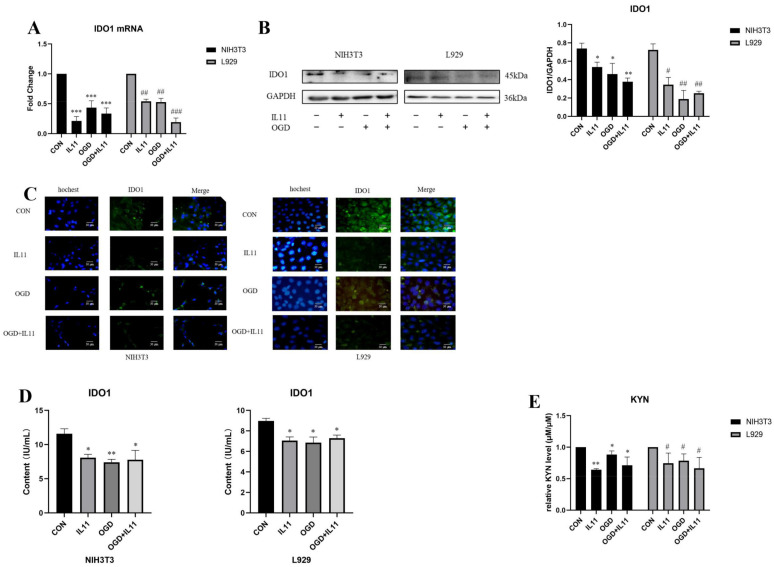
**IL11 inhibited the expression of IDO1.** (**A**) mRNA expression estimated by RT-PCR for IDO1 after supplementing rmIL11 in NIH3T3 and L929 cells. (**B**) Western blot analysis and densitometric analysis of IDO1 protein levels after supplementing rmIL11 in NIH3T3 and L929 cells; GAPDH was used as the loading control. (**C**) Representative immunocytochemistry staining of IDO1 and cell nuclei (Hoechst) after supplementing rmIL11 in NIH3T3 and L929 cells. (**D**) IDO1 content estimated by ELISA from treated cell lysate. (**E**) The relative KYN level was detected by Ehrlich assay after inhibiting IL11 in NIH3T3 and L929 cells. OGD—oxygen–glucose deprivation; CON—control. The results are presented as the means ± SDs, and Student’s t-test was used to determine the *p*-value. * *p* < 0.05, ** *p* < 0.01, and *** *p* < 0.001, compared with CON group in NIH3T3 cells; ^#^ *p* < 0.05, ^##^ *p* < 0.01, and ### *p* < 0.001 compared with CON group in L929 cells.

Recombinant IL11 was administered to overexpress IL11, and the changes in IDO1 were detected by RT-PCR, Western blot, and immunofluorescence. It was found that IDO1 mRNA (Figure 9A) and protein expression levels were down-regulated in NIH3T3 and L929 cells under OGD conditions (Figure 9B,C). The ELISA kit assay results show that after the high expression of IL11, the content of IDO1 in NIH3T3 and L929 cells decreased under OGD conditions (Figure 9D). The Ehrlich test results show that after the overexpression of IL11, KYN expression decreased in NIH3T3 and L929 cells under OGD conditions (Figure 9E). The above results show that OGD inhibited the expression of IDO1/KYN in fibroblasts based on IL11.

**Figure 10:**
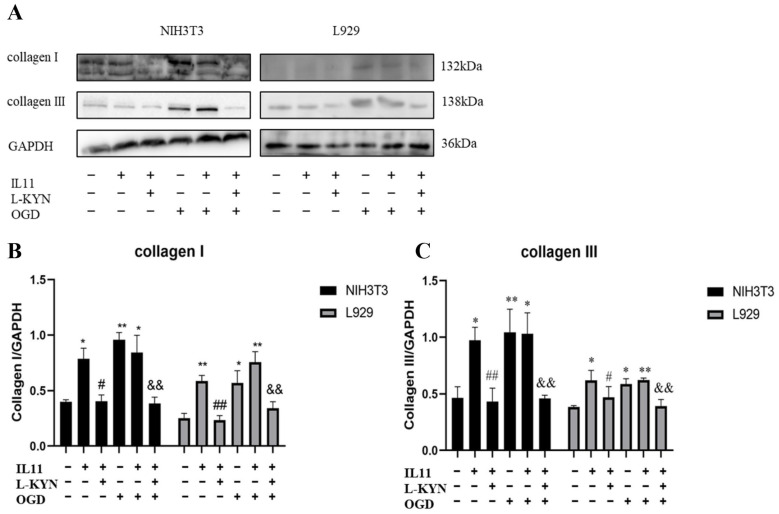
**OGD inhibits the expression of IDO1/KYN in fibroblasts via IL11.** Western blot analysis and densitometric analysis of collagen I and collagen III protein levels after supplementing 50 μM KYN and 25 ng/mL rmIL11 in NIH3T3 and L929 cells; GAPDH was used as the loading control. OGD—oxygen–glucose deprivation; CON—control. The results are presented as the means ± SDs, and Student’s t-test was used to determine the *p*-value. * *p* < 0.5 and ** *p* < 0.01, compared with CON group; ^#^ *p* < 0.05 and ^##^ *p* < 0.01, compared with IL11 group; ^&^ *p* < 0.05 and ^&&^ *p* < 0.01, compared with IL11 + OGD group.

Our group’s previous experiments determined that IL11 inhibits the expression of fibroblast senescence-related proteins based on IDO1/KYN. To determine the effect of IL11 based on IDO1/KYN on collagen-related proteins in fibroblasts, we administered IL11 and KYN at the same time to detect the protein expression levels of collagen I and collagen III in NIH3T3 and L929 cells induced by OGD. Compared with the supplementation with IL11, supplementation with IL11 and KYN resulted in the down-regulation of collagen I and III protein expression in NIH3T3 and L929 cells (Figure 10). These results indicate that the increase in KYN inhibited the promoting effect of IL11 on collagen I and III protein expression. In conclusion, it is suggested that IL11 may promote collagen expression in fibroblasts under oxygen–glucose deprivation by affecting IDO1.

## 3. Discussion

Fibroblasts play an important role in the repair of tissue ischemia injury, and ischemia-induced fibroblast senescence affects the final result of the repair. Consistent with previous studies, OGD can simulate ischemic stress, serve as an in vitro model of myocardial ischemia and stroke, and promote cell senescence [16,17]. NIH3T3 cells from mouse embryonic connective tissue and L929 cells from mouse dermal connective tissue are commonly used to study ischemic injury and cell senescence [18,19]. By comparing these two fibroblast lines, we found that NIH3T3 and L929 cells can undergo stress-induced senescence induced by OGD, which is manifested by the increased expression of the p53, p21, and p16 proteins, as well as the increased expression of collagen. However, some studies have found that the expression of type I collagen in fibroblasts in senescent skin is decreased [20], suggesting that the changing trend of collagen expression in senescent fibroblasts is different. Because type I collagen is also the target of time and light senescence, these will promote the degradation and decrease the yield of this protein, which also shows that the expression of type I collagen is regulated by many factors. The different functions of senescent cells induced by different external induction conditions will lead to different collagen expressions. Senescent cells induced by stress such as ischemia only exist briefly in the process of tissue repair to accelerate wound healing. However, senescent cells that persist in senescent or chronically damaged tissues or cells with replicative senescence caused by a continuous passage in vitro tend to reduce collagen secretion and increase matrix metalloproteinase secretion. The mechanisms and functions of these two types of senescent cells are different. In addition, the functional differences of fibroblasts from different sources will also lead to differences. For example, the senescence of skin fibroblasts can lead to a reduction in collagen, which is related to the replication senescence induced by telomere shortening and the increase in extracellular matrix metalloproteinases induced by ultraviolet light. However, some studies have found that the early stage of liver injury benefits from the increase in collagen in senescent fibroblasts. However, with the continuous activation of senescent fibroblasts, the secretion of SASP factors increases, which intensifies the inflammatory response and ultimately affects the healing of the injured area. This also suggests that OGD induces stress-induced senescence in NIH3T3 and L929 cells, activates fibroblasts, and induces increased collagen expression. Ischemia can induce fibroblasts to secrete a large amount of collagen and promote repair. However, studies have found that fibroblasts continue to activate after stress-induced senescence, leading to the deposition of the extracellular matrix and senescent tissue function [11]. In addition, the expression of p53 and p21 in senescent fibroblasts is up-regulated, which promotes the release of pro-inflammatory factors, intensifies the inflammatory response, and finally aggravates the ischemic injury. Therefore, our results elucidate the regulatory mechanism of fibroblast senescence after ischemia and could provide an effective basis for the treatment of ischemic diseases.

IL11 is a member of the IL6 cytokine family, mainly derived from stromal cells such as fibroblasts [8]. In fibroblasts, IL11 signaling activates JAK/STAT3 and MEK/ERK via IL11RA/IL6ST and possibly AKT [21]. MEK/ERK activity correlates with the pro-fibrotic effect of IL11 [22]. IL11 may trigger an inflammatory cascade. A recent study reported that IL11 triggered a pro-inflammatory secretome with the notable up-regulation of IL8, IL6, MCP1, CCL20, and CXCL1/5/6, which are important chemotaxins for neutrophils, monocytes, and lymphocytes [23]. IL11 is a multifunctional cytokine produced by fibroblasts and other stromal cells, which play an important role in cell senescence. In the study of lung fibroblast senescence, the TGFβ/IL11/MEK/ERK signaling pathway can promote fibroblast senescence and collagen secretion [11]. IL11 is also an important component of SASP factors, which suggests that IL11 can regulate ischemia-induced stress-induced senescence of fibroblasts [24]. In cardiac fibroblasts, supplementation of IL11 can effectively activate downstream signal pathways and promote fibrosis [22]. Therefore, the present study investigated the effects of overexpression of IL11 on OGD-induced senescence and collagen expression in fibroblasts by supplementing IL11 and activating downstream signaling pathways. By inhibiting IL11 [25,26] and supplementing IL11 [27], it was found that OGD promoted fibroblast senescence and collagen expression via IL11.

Prolonged ischemia and hypoxia can lead to the fluctuation and imbalance of energy metabolism. A large number of studies have found that the enhancement of KYN metabolism is related to tissue and cell ischemia [28] and affects ischemia-induced cell senescence [29]. During ischemia injury, fibroblasts participate in cell repair by expressing and secreting collagen, while KYN can inhibit the collagen expression of fibroblasts by activating AhR. In this study, the expression changes of IDO1 in NIH3T3 and L929 fibroblasts induced by OGD were elucidated for the first time in a variety of ways, and the relationship between IL11, IDO1, and its mediated kynurenine metabolites in OGD-promoted fibroblast senescence and collagen expression was preliminarily elucidated. This provides a theoretical basis for the further study of IDO1 and kynurenine metabolism in the OGD-induced stress-induced senescence of fibroblasts and the mechanism of IL11 regulation of IDO1 and kynurenine metabolism. In addition, our previous work found that IL11 induces the expression of stress-related senescence proteins in NIH3T3 and L929 fibroblasts based on the IDO1/KYN metabolic pathway. Therefore, we found that KYN was down-regulated and collagen expression was increased after OGD-promoted fibroblast senescence, and IL11 inhibition of IDO1 expression might be the reason for this.

**Figure 11:**
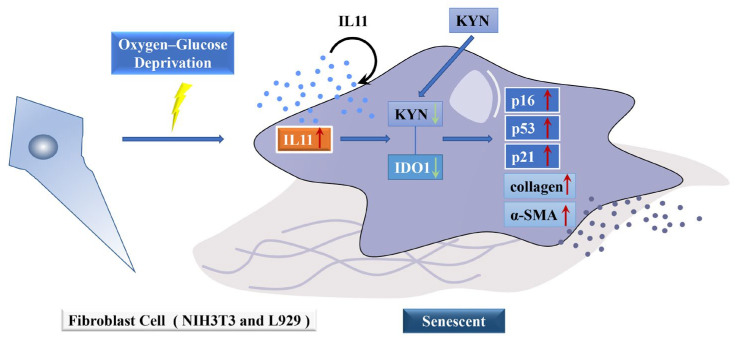
**A schematic diagram of the mechanism by which OGD promotes senescence and collagen expression in fibroblasts.** We found that OGD may promote fibroblast senescence and collagen expression through IL11 inhibition of the IDO1/KYN metabolic pathway.

Overall, our study established that OGD can promote senescence and collagen expression in fibroblasts, and this effect is mediated by IL11. We found that OGD may affect the IDO1/KYN metabolic pathway through IL11, thereby affecting fibroblast senescence and collagen expression, which may provide an effective basis for the treatment of cellular senescence caused by ischemic disease (Figure 11).

## 4. Materials and Methods

### 4.1. Cell Culture and Stimulations

NIH3T3 and L929 cells were obtained from Shanghai Zhong Qiao Xin Zhou Biotechnology Co.Ltd. The cells were cultured at 37 °C in a 5% CO_2_ atmosphere using RPMI Medium 1640 basic (1640; Gibco, New York, NY, USA) supplemented with 10% fetal bovine serum (FBS, Biological Industries, Brisbane, BI, Australia), 1:100 penicillin/streptomycin, and 100 mM nonessential amino acids(Gibco, USA). Cells were passaged using a standard protocol with 0.25% trypsin (Sigma, Saint Louis, MO, USA) and seeded 12 h before experiments. The cells were seeded in 6-well culture plates at a confluence of 50%. The cells were stimulated with 25 ng/mL IL11 (GenScript, Nanjing, China) and/or 50 µM L-KYN (Macklin, Shanghai, China) diluted with RPMI 1640 for 2 h. For hypoxic conditions, the cells were grown in a three-gas incubator (SANYO) with 1% oxygen, 5% carbon dioxide, and 94% nitrogen. For glucose deprivation conditions, PRMI-1640 medium without glucose (Gibco, USA) was used.

### 4.2. Antibody and Reagents

Collagen I antibody (AF7001, 1:1000), collagen III antibody (AF0136, 1:1000), α-SMA antibody (AF1032, 1:1000), p53 antibody (AF0879, 1:1000), p21 antibody (AF6290, 1:1000), and p16 antibody (AF5484, 1:1000) were purchased from Affinity Biosciences. IL11 antibody (A1902) was purchased from ABclonal. rmIL11 (Z03052) was purchased from GenScript, and L-KYN (L864410) was purchased from Macklin.

### 4.3. Cell Transfection

Small interfering RNA targeting IL11 (si-IL11) (CACAGATGAGAGACAAATT) was purchased from RIBOBIO. siRNAs (si-IL11 and si-NC) were transfected into NIH3T3 and L929 cells with a final concentration of 20 nM using Lipofectamine 2000 (Invitrogen, Waltham, MA, USA) according to the manufacturer’s instructions. After being transfected for 24 h, cells were subjected to OGD for 6 h. Cells were then harvested for the subsequent experiments.

### 4.4. RNA Isolation and Quantitative RT-PCR

Cultured cells were treated with Trizol reagent (Thermo Fisher, USA). The cDNA Synthesis Kit (TransGen Biotech, Beijing, China) was used for reverse transcription PCR (RT-PCR). Comparative quantitative PCR (Q-PCR) was performed by using the SYBR Green Q-PCR Kit (Roche, Mannheim, Germany). Primers are listed as follows. The Ct values were analyzed using the ΔΔCt method, and relative changes in mRNA levels were obtained by normalization to GAPDH relative to the control. IDO1: 5′-AGGATGCGTGACTTTGTG-3′ and 5′-GAGGGCTCTTCCGACTT-3′; α-SMA: 5′-CATCAGGGAGTAATGGTTGGAATGGG-3′ and 5′-GTGTTCTATCGGATACTTCAGCGTCAG-3′; IL11: 5′-AGGTGGTCCTTCCCTAAAGACTCTG-3′ and 5′-CAAGAGCTGTAAACGGCGGAGTAG-3′; collagen III: 5′-AGTCGGAGGAATGGGTGGCTATC-3 and 5′-CAGGAGATCCAGGATGTCCAGAGG-3′; collagen I: 5′-GGGCAACAGCAGATTCACCTACAC-3′ and 5′-CAAGGAATGGCAGGCGAGATGG-3′; GAPDH: 5′-AAGCCCATCACCATCTTCCA-3′ and 5′-CCTGCCTCACCACCTTCTTG-3′. 

### 4.5. Western Blot

The protein lysate from cells was isolated using RIPA (Solarbio) with protease and phosphatase inhibitors for 10 min and then centrifuged for 20 min, 4 °C, 14,000× *g*. After 30 min of incubation on ice, the lysates were centrifuged for 20 min, 4 °C, 10,000× *g*. The electrophoresis separation was carried out in 10% polyacrylamide gel. After the transfer, PVDF (Polyvinylidene fluoride) membranes (Merck-Millipore, Burlington, MA, USA) were blocked for 1 h in 5% milk in Tris-buffered saline with 0.1% Tween (TBS-T) followed by overnight incubation in the primary antibody at 4 °C. On the following day, the membranes were rinsed with TBS-T and incubated for 1 h with the secondary antibody. Chemiluminescent HRP Substrate (Merck-Millipore) and the ChemiDoc system (Bio-Rad, Hercules, CA, USA) were used for signal detection. Analysis was conducted in three independent experiments.

### 4.6. ELISA

The lysates for ELISA were isolated from cells in 200 μL precooled PBS (containing protease inhibitor), followed by ice bath ultrasound for 3 min and centrifugation at 4 °C, 12,000× *g* for 10 min. To evaluate levels of IDO1 in fibroblasts, ELISA assays were performed (YX-090415M, Sinobestbio) according to the manufacturer’s instructions.

### 4.7. Collagen Contraction Assay

After stimulation, cells were trypsinized, counted, and mixed with the Collagen Gel Working Solution prepared according to the manufacturer’s protocol (C8062, Solarbio). The solution was added to 24 well plates and allowed to polymerize for 20 min at 37 °C. Fresh growth medium was added to the solidified collagen gels, and plates were returned to the incubator. After OGD for 6 h, the surface of contraction was released and contraction was monitored. Next, the surface area of contracted gels was imaged using a Chemi Doc instrument (Bio-Rad) and measured using the ImageJ software (NIH).

### 4.8. Immunofluorescence

After stimulation, cells were fixed with 4% paraformaldehyde for 5 min at room temperature, permeabilized with 0.01% Triton X-100 for 5 min, and blocked with 5% BSA in PBS for 20 min. Specimens were incubated with primary mouse anti-IDO1 (1:500, clone CAT-5H10, Thermo Fisher Scientific, Waltham, MA, USA) and an anti-αSMA antibody (1:200) at room temperature, followed by the secondary AlexaFluor555 goat anti-rabbit antibody (1:8000) at room temperature for 1 h. Hoechst 3342 (1:1000) was used to label the nuclei. Immunofluorescence was analyzed using a ZEISS Axio Scope5/ A1 fluorescence microscope.

### 4.9. MTT Assay

Under OGD conditions for 2 h, 4 h, 6 h, 8 h, and 10 h, 20 μL of 5 mg/mL MTT solution was added to each well, and the plate was further incubated at 37 °C for 4 h. Thereafter the medium was aspirated, the wells were washed with PBS and allowed to dry for approximately 2 h, and 200 μL of DMSO was added to each well. The microtiter plate was placed on a shaker to dissolve the dye. After the formazan crystals had dissolved, the absorbance was determined at 490 nm with a fluorescence microplate reader.

### 4.10. Ehrlich Assay

After treating cells, 1 mL of the cell supernatant was collected. Then 100 μL of 30% TCA solution was added to each tube of samples and standards and mixed upside down at 65 °C for 15 min, and then 12,000× *g* for 10 min. The supernatant was collected, and 140 μL was added to each well of the 96 well plates. An equal volume of 2% 4-dimethylaminobenzaldehyde was added per hole; the absorbance was determined at 490 nm with a fluorescence microplate reader. The standard curve (12, 6, 3, 1.5, 0.75, 0.375, 0.1875, and 0.09375 μM) was plotted. According to the standard curve, the corresponding concentration and the ratio of the treatment group to the control group were calculated.

### 4.11. Statistical Analysis

The in vitro experiments were performed in three independent trials, and all the results are presented as the means ± SDs. An unpaired Student *t*-test was used for comparison of the two groups: * p < 0.05; ** p < 0.01; *** p < 0.001.

## Data Availability

Not applicable.
